# Effect of Concentration of Thermochromic Ink on Performance of Waterborne Finish Films for the Surface of Cunninghamia Lanceolata

**DOI:** 10.3390/polym12030552

**Published:** 2020-03-03

**Authors:** Xiaoxing Yan, Yijuan Chang, Xingyu Qian

**Affiliations:** College of Furnishings and Industrial Design, Nanjing Forestry University, Nanjing 210037, China; changyijuan@njfu.edu.cn (Y.C.); qianxingyu@njfu.edu.cn (X.Q.)

**Keywords:** thermochromic ink, finish film, film performance

## Abstract

Using Cunninghamia lanceolata as a substrate, the thermochromic ink was added to the waterborne finish to test the optical properties and mechanical properties of the finish film. The results showed that the discoloration performance of the finish film with 15.0% and 30.0% of the thermochromic ink was better. The gloss of the finish film changes irregularly when the concentration increases. The finish film with a thermochromic ink concentration of 10.0% has the highest gloss, and with a concentration of 30.0% has the lowest gloss. When the thermochromic ink concentration exceeds 15.0%, the impact resistance of the finish film is slightly enhanced. The concentration is not related to the liquid resistance of the finish film. When the thermochromic ink concentration was 0–15.0%, the particle distribution uniform reunion was not much. The discoloration mechanism of discolored finish film can be considered to be as follows. After adding thermochromic ink, when the finish film temperature rises, it fades from red to colorless. When the temperature is lowered, the thermochromic ink changes to its original colour again, and the thermochromic effect is stable and sustainable. On the basis of the above results, when the thermochromic ink concentration is 15.0%, the general performance of the waterborne finish film on the Cunninghamia lanceolata surface is the best. This study provides new prospects in using thermochromic ink for waterborne finish film.

## 1. Introduction

Temperature-sensitive reversible thermochromic ink is a kind of discoloring material that changes with temperature. It can show different colour effects with the the change of temperature [1]. The reversible temperature-changing thermochromic ink has the advantages of convenient detection, fast, accurate, and no need for additional auxiliary equipment, and has broad application prospects in intelligent printing [2]. Organic reversible temperature-changing thermochromic materials have remarkable characteristics in terms of colour change temperature, thermochromic colour combination, colour contrast before and after discoloration, etc., and are the most promising reversible temperature-changing thermochromic materials [3,4]. The organic reversible thermochromic material is mainly composed of a coloring agent, a colour developing agent and a solvent. In order to reduce the influence of other side reactions and environmental factors in the process of discoloration, microencapsulation treatment of temperature-changing thermochromic materials is often carried out to expand the application range of temperature-changing material systems [5].

Yan et al. [6] added glass fiber powder to the surface of the Chinese fir with thermochromic ink to investigate the heat-insulating effect of glass fiber powder. The results showed that when 3.0% glass fiber powder was added, the comprehensive performance was optimal. Hanzer et al. [7] analyzed three offset inks, one that dries by absorption and two that dry by oxypolymerization of vegetable oils. The results showed that the deinked paper contained bisphenol A, but does not release toxic substances. Cho et al. [8] used molecular printing to print thermochromic inks in cyan, magenta, yellow, and black colours and integrated them into thermochromic device prototypes for potential display applications. Vinkovic et al. [9] invented a new means for analyzing bisphenol A and benzophenone in thermochromic printing inks to identify inks containing these hazardous materials. The results showed that ultrasonic-assisted methanol extraction was appropriate and effective.

Environmentally friendly waterborne finish uses water as a solvent to save a large number of resources and protect the atmosphere [10]. A waterborne finish has good adaptability to the surface of the material and strong adhesion of the coating [11]. The thermochromic ink is added to the waterborne finish, so that the waterborne finish changes colour according to the change of temperature, thereby forming a new type of smart paint [12,13]. The material of Cunninghamia lanceolata is light, easy to dry, small shrinkage, no curling, good durability, easy processing, and has excellent bonding performance [14]. Finish film refers to the coating on the upper layer of the film, which requires good decorative performance and unique physical and chemical properties, so as to better protect and beautify the wood base material such as Cunninghamia lanceolate [15].

In this paper, Cunninghamia lanceolata was used as the base material and the waterborne finish was used as the base, the thermochromic ink was added to the waterborne finish, and the influence of the concentrationration of the thermochromic ink on the performance of the waterborne finish film on the Cunninghamia lanceolata surface, such as colour difference, gloss, adhesion, and liquid resistance was analyzed. The best thermochromic ink concentration for the performance of the finish film was explored, which provides reference for the application of colour-changing film on wood surface.

## 2. Materials and Methods

### 2.1. Experimental Materials

Methyl red (as leuco agent, *M*_w_: 269.3 g/mol, CAS No.: 493-52-7), bisphenol A (as colour developer, *M*_w_: 228.29 g/mol, CAS No.: 80-05-7), tetradecanol (*M*_w_: 214.39 g/mol, CAS No.: 103-20-8) and melamine (*M*_w_: 126.12 g/mol, CAS No.: 108-78-1) were supplied by Huancai Discoloration Technology Co., Ltd., Shenzhen, China. A 37.0% formaldehyde solution (*M*_w_: 30.03 g/mol, CAS No.: 50-00-0), anhydrous ethanol (*M*_w_: 46.07 g/mol, CAS No.: 64-17-5), sodium dodecyl benzene sulfonate (*M*_w_: 348.48 g/mol, CAS No. 25155-30-0), citric acid monohydrate (*M*_w_: 210.14 g/mol, CAS No.: 5949-29-1) and triethanolamine (*M*_w_: 149.19 g/mol, CAS No.: 102-71-6) were supplied by Shanpuhuagong Co., Ltd., Shanghai, China. The waterborne wood finish was supplied by Yihua Lifestyle Technology Co., Ltd., Shantou, China. Waterborne coating consists of waterborne acrylic copolymer dispersion (the concentration was 90.0%), matting agent (the concentration was 2.0%), additives (the concentration was 2.0%) and water (the concentration was 6.0%). The 15.0% NaCl solution and 70.0% medical ethanol were provided by Yueyang Baling Huaxing Petrochemical Co., Ltd., Yueyang, China. Detergent was supplied by Hutchison WhiteCat Co., Ltd., Shanghai, China. Red ink was supplied by Fine Stationery Co., Ltd., Shanghai, China. Cunninghamia lanceolata boards (100 mm × 100 mm × 12 mm, uniform material colour, 300 pieces, after ordinary mechanical sanding) were supplied from Yihua Lifestyle Technology Co., Ltd., Shantou, China.

### 2.2. Preparation of the Thermochromic Ink

Thermochromic ink was prepared by emulsion polymerization. A total of 5.0 g of melamine, 10.0 g of 37.0% formaldehyde solution, and 10.0 mL of deionized water were poured into the beaker. The pH was adjusted to 8.5–9.0 with triethanolamine. Then, the beaker was placed in a 70 °C thermostatic water bath with a stirring rate of 700 rpm, and after stirring for 1 h, a transparent solution was obtained as a wall material.

The 1.0 g of sodium dodecyl benzene sulfonate was completely dissolved in 99.0 g of deionized water to obtain a 1.0% emulsifier solution. Then, a 1.0% emulsifier solution was added to a mixed solution of 1.0 g of methyl red, 1.0 g of bisphenol A, and 10.0 g of tetradecanol, and the beaker was placed in a 60 °C water bath. Stirring was continued at 1200 rpm for 30 min to obtain a stable core emulsion.

The core emulsion was added to the wall material solution and stirred uniformly. The pH of the solution was adjusted to 4.0–5.0 by adding citric acid monohydrate. The mixed solution was reacted at 60 °C for 3 h, and then cooled to room temperature. After aging for 5 days, it was filtered with a vacuum suction filter bottle, washed with deionized water and anhydrous ethanol for several times, and the powder was placed in an oven and dried at 25 °C for 24 h to obtain 6.0 g of thermochromic microcapsules. The thermochromic microcapsules were added to 10.0 g of an aqueous acrylic copolymer dispersion and uniformly mixed to obtain a thermochromic ink.

### 2.3. Preparation of Coatings

A sample of 50.0 g of thermochromatic waterborne finish coating was prepared, and the thermochromic ink concentration was 0, 5.0%, 10.0%, 15.0%, 20.0%, 25.0%, and 30.0%, respectively. The composition is shown in Table 1. The finish coating was applied on Cunninghamia lanceolata boards with SZQ tetrahedral fabricator (Tianjin Jinghai science and technology testing machinery factory, Tianjin, China). After the surface was dried for about 30 min, the sample was moved to a 30 °C electric heating oven, heated until the quality did not change, and then taken out, and naturally cooled to room temperature. Then, 1000 mesh sandpaper was used to lightly polish the coating surface and dry cloth to remove floating dust. The finish was applied two more times as described above. The thickness of the prepared dry finish film was about 60 µm.

### 2.4. Testing and Characterization

The colour difference of the finish film from 18 to 40 °C was measured using a SEGT-J portable colorimeter (Zhuhai Tianchuang Instrument Co., Ltd., Zhuhai, China). The coating was heated on a HHP1 heating plate (Shanghai Hengyue Medical Devices Co., Ltd., Shanghai, China). The coating was heated slowly, the surface temperature of the coating was measured with a temperature sensor, and the chroma value of the coating was measured with a colorimeter to determine the temperature dependence of the result. The gloss, impact resistance, and adhesion of finish films were measured according to reference [16]. The gloss of the finish film was tested using a 3nh smart gloss meter (produced by 3NH Technology Co., Ltd., Shenzhen, China). The adhesion of the finish film was tested by QFZ-II coating adhesion tester (Tianjin JingKelian Material Testing Machine Co., Ltd., Tianjin, China). The impact resistance of finish film was tested by QCJ-50 finish film impactor. The weight of the hammer is 1000 ± 1 g, the total height is 50 cm, and the minimum scale is 1 cm. The impact resistance of the coating is indicated by the maximum height of the hammer which will not cause damage when the hammer falls on the film. The liquid resistance of the coating was measured using a 15.0% NaCl solution, 70.0% of medical ethanol, detergent, and red ink. The microstructure of the finish film was tested by Quanta 200 environment scanning electron microscope (SEM), FEI Company (Hillsboro, OR, USA). The chemical composition of the finish film was tested by a VERTEX 80v infrared spectrometer, Germany BRUKER Co., Ltd., Karlsruhe, Germany [17]. The ageing and stability test was measured in a ZN ultraviolet weather resistance tester (Nanjing Environmental Test Equipment Co., Ltd., Nanjing, China). A mixture of rosin and paraffin was coated on the side and the back of Cunninghamia lanceolata boards, leaving a 10.0 × 5.0 cm^2^ coating, and the samples were put in the ZN ultraviolet weather resistance tester for 240 h. The UV region was considered as 290–400 nm and the illumination level was 0.08 W/cm^2^. All the experiments were repeated four times with an error of less than 5.0%.

## 3. Results and Discussion

### 3.1. Effect of Thermochromic Ink Concentration on Colour Difference

The waterborne finish film with different thermochromic ink concentration was heated from 18 to 40 °C, and the chroma values of the film were recorded every two degrees. L* represents brightness. If L* is positive, it means that the coating surface is bright, and if L* is negative, it means that the coating surface is dark. “a*” indicates red green colour, a positive number indicates red colour, and a negative number indicates green colour. "b*" indicates the yellow blue colour. If “b*” is a positive number, the coating surface to be tested is yellow. If “b*” is a negative number, the coating surface to be tested is blue. “C” expresses the saturation. The “H” means hue. The colour difference (ΔE) was calculated according to Formula (1): [18]

**Figure 1:**
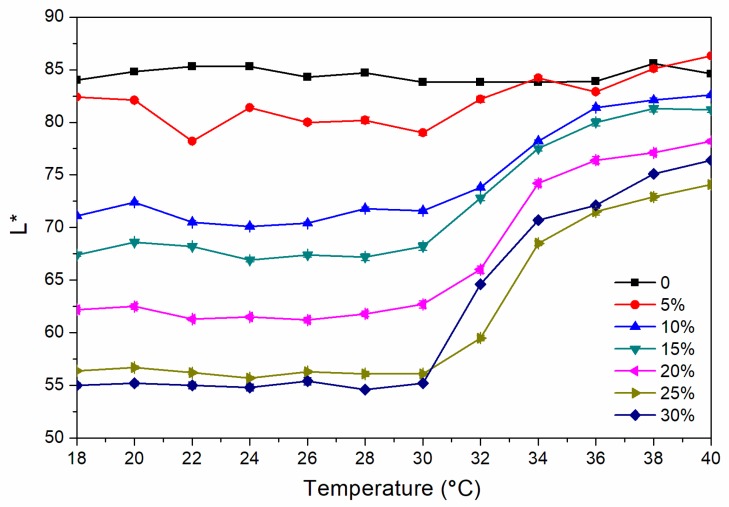
Change of L* value from 18 to 40 °C.

**Figure 2:**
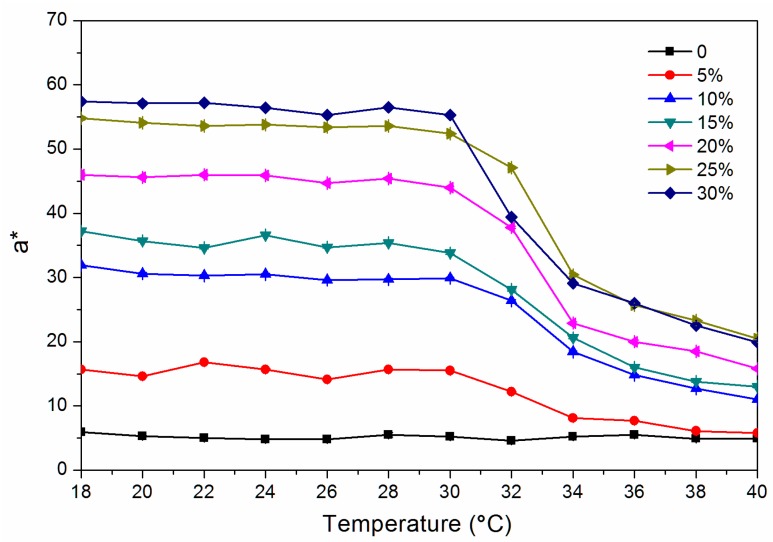
Change of a* value from 18 to 40 °C.

**Figure 3:**
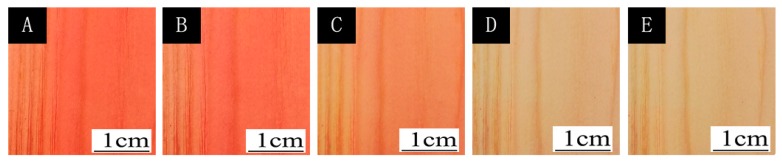
*Image of of the finish film with 15.0% thermochromic ink changing with temperature*: (**A**) 20 °C, (**B**) 32 °C, (**C**) 34 °C, (**D**) 36 °C, (**E**) 40 °C.

ΔE=[(ΔL*)^2^+(Δa*)^2^+(Δb*)^2^]^1/2^ (1)

where ΔL* (lightness difference) = L_1_* − L_2_*, Δa* (red and green difference) = a_1_* − a_2_*, Δb* (yellow blue difference) = b_1_* − b_2_*. Figure 1 and Figure 2 show the chroma value image of L* and a* of the finish film with different concentration of thermochromic ink from 18 to 40 °C. The a* value changed obviously with the temperature, which showed that the red green colour of the finish film changed significantly. Figure 3 shows the image of the finish film with 15.0% thermochromic ink changing with temperature, and the colour of finish film changing from red to colorless.

**Figure 4:**
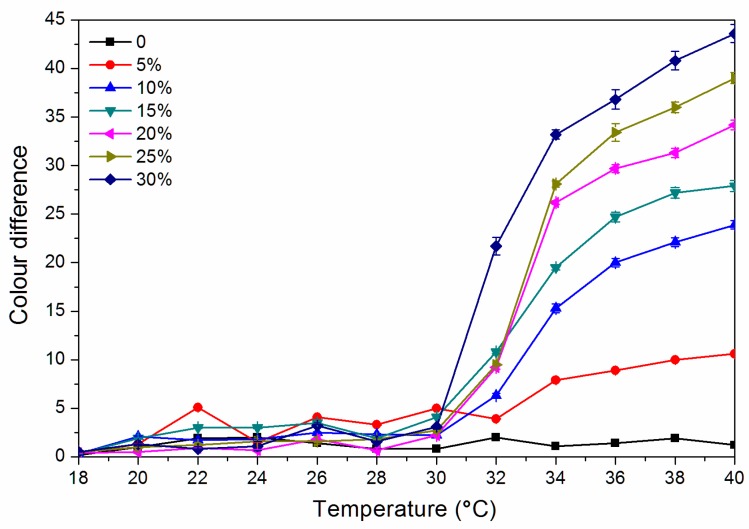
Effect of thermochromic ink concentration on colour difference of waterborne finish film from 18 to 40 °C.

**Figure 5:**
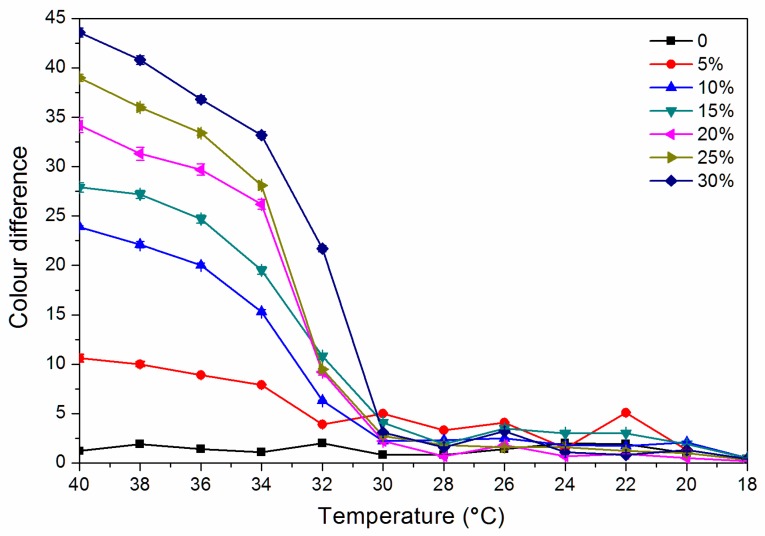
Effect of thermochromic ink concentration on colour difference of waterborne finish film from 40 to 18 °C.

As shown in Figure 4, the colour difference of the finish film with a thermochromic ink concentration of 0% from 18.0 °C to 40.0 °C is 0.8–2.0, and there is no thermochromic effect. The thermochromic ink of 5.0%–30.0% of the finish film at 18.0–30.0 °C has a colour difference of 0.5–5.0, and the colour shows no significant change. When the finish film with the thermochromic ink concentration of 30.0% is heated to 32.0 °C, the thermochromic is most remarkable, and the finish film has a colour difference of 21.7 while that with a concentration of 15.0% has a colour difference of 10.8 at 32 °C. Figure 5 shows the cooling colour difference from 40 to 18 °C, which implied that the finish film has good colour changing ability. Therefore, a finish film with a large colour difference around 30 °C has a better discoloration effect. In summary, it is preliminarily believed that the finish film with a thermochromic ink concentration of 30.0% and 15.0% has a better colour-changing effect.

### 3.2. Gloss Analysis

The finish films were irradiated with 20°, 60°, and 85°, respectively. Table 2 indicates that when the same thermochromic ink concentration is added, as the incident angle increases, the gloss of the finish film increases. Under the same intensity of incident light, as the concentration of the thermochromic ink increases, the gloss of the finish film changes irregularly. The gloss of the finish film with a concentration of 10.0% is the highest, and the gloss of the finish film with a concentration of 30.0% is the lowest.

### 3.3. Effect of Thermochromic Ink Concentration on Adhesion and Impact Resistance of Finish Film

The lower the adhesion level, the smaller the area where the coating is damaged, and therefore, the better the adhesion. It can be seen from Table 3 that the adhesion level of the finish film with a thermochromic ink concentration of 0%–30.0% is 0, which is good, so the concentration of the thermochromic ink has no effect on the adhesion of finish film. As shown in Table 3, the impact resistance of the film without thermochromic ink was 40.0 N cm^−1^. When the concentration increases from 5.0% to 30.0%, the impact resistance of the finish film is gradually increased.

### 3.4. Effect of Thermochromic Ink Concentration on Liquid Resistance

The four kinds of test liquids for the liquid resistance test of finish film with the concentration of thermochromic ink of 0%–30.0% is 15.0% NaCl solution, detergent, red ink and 70.0% medical ethanol. When the temperature was set at 18 °C, the colour values of the finish film were observed before the experiment and 24 h later, and the colour difference was calculated (Table 4). As shown in Table 5 and Table 6, the finish film with a thermochromic ink concentration of 0%–30.0% has a liquid resistance level of 1 for all the test liguids, and has no mark and good liquid resistance. It indicates that the thermochromic ink concentration has no effect on the liquid resistance level of the finish film.

### 3.5. Microstructure Analysis

**Figure 6:**
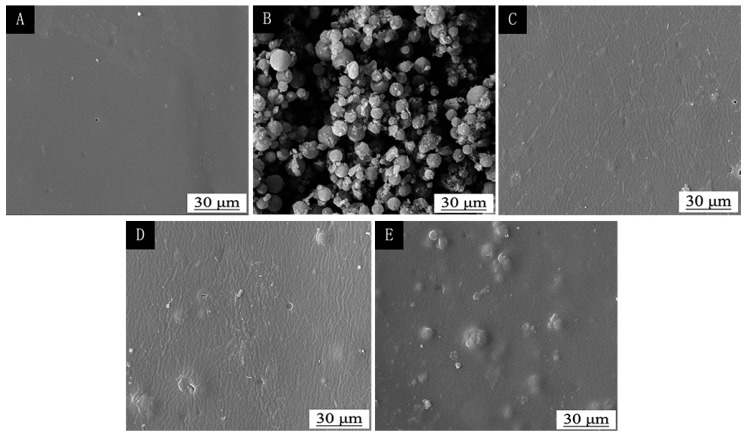
SEM image of (**A**) pure finish film and (**B**) thermochromic microcapsules. The finish film with different thermochromic ink concentration: (**C**) 5.0%, (**D**) 15.0%, and (**E**) 30.0%.

As shown in Figure 6, the pure finish film has a smooth surface with almost no particles. The finish with 5.0% thermochromic ink appeared in granules, but the particles were less. The finish with 15.0% thermochromic ink had obvious granules, the particle distribution was relatively uniform, and there was less agglomeration. The finish with 30.0% thermochromic ink was obviously agglomerated. Therefore, with the increase of the concentration of the thermochromic ink, the thermochromic microcapsules in the film are more likely to agglomerate. The microcapsules in the film with 0%–15.0% thermochromic ink are not easy to agglomerate, the finish film is smooth and flat, and the microstructure of the finish film is good.

### 3.6. Stability of Thermochromic Finish Film

Based on the above optical properties, mechanical properties, liquid resistance, and SEM results, the overall performance of the finish film is best when the thermochromic ink concentration of the finish is 15.0%. When the indoor temperature in summer is about 30 °C, people usually feel hot. The thermochromic finish film has a better colour-changing effect near 30 °C, so the effect of time on the colour difference with the optimal thermochromic ink concentration (15.0%) has been tested at 30 °C. The discoloration performance of 15.0% thermochromic finish film after three months was tested, and the colour difference was calculated. Table 7 shows that the colour difference of the finish film at room temperature and at 30 °C for three consecutive days after three months is 1.3–1.7, and has no obvious colour change, indicating that time has no effect on the colour change performance of the best thermochromic ink concentration of the finish film.

**Figure 7:**
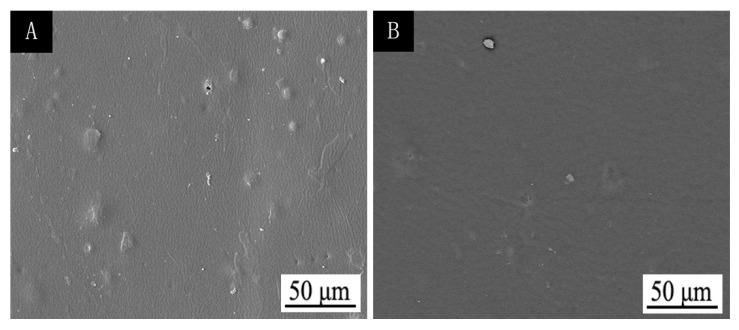
SEM of finish film with 15.0% thermochromic ink before (**A**) and after (**B**) UV aging.

After ultraviolet-accelerated aging (Table 8), the colour difference was only 3.5, and the gloss was slightly reduced. As shown in Figure 7, the finish film after UV aging is smooth and flat, which indicates that the discoloration effect of thermochromic finish film is stable and the discoloration effect is better.

**Figure 8:**
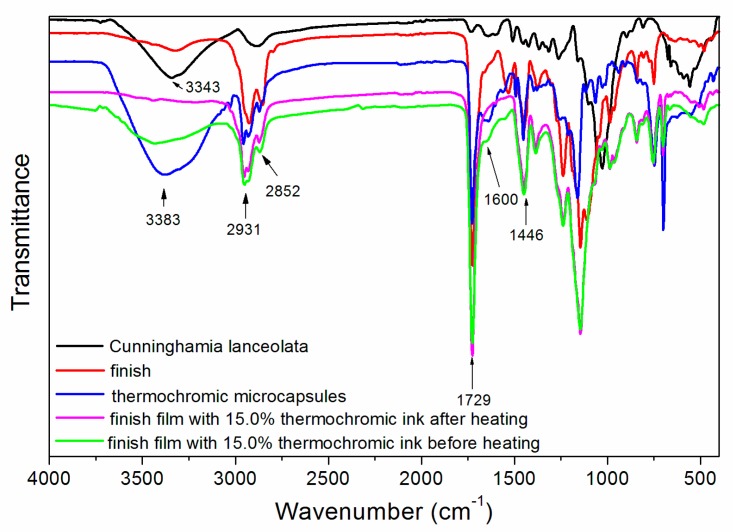
Infrared spectra of Cunninghamia lanceolata, waterborne wood finish, thermochromic microcapsules, finish film with 15.0% thermochromic ink before and after heating.

Infrared spectroscopy tests were carried out on a waterborne wood finish, thermochromic microcapsules, Cunninghamia lanceolata, and finish film with with 15.0% thermochromic ink before and after heating, as shown in Figure 8. The attribution of peaks is shown in Table 9. As can be seen from Figure 8 and Table 9, 3383 and 3343 cm^−1^ were the –OH absorption peaks [19]. The 2931 and 2852 cm^−1^ peaks represent –CH_2_– asymmetric and symmetric stretching vibrations. 2931, 2852 and 1446 cm^−1^ were –CH_3_ absorption peaks, and 1729 cm^−1^ was a strong and sharp characteristic peak of the C=O group. The absorption peaks of C=C and N=N appeared near 1600 cm^−1^. When the colour of the microcapsules changes, COOH may change to COO– [20], and the nitrogen–nitrogen double bond (N=N) may be broken [21]. Furthermore, the absorption peaks of the finish film with 15.0% thermochromic ink before and after heating were not damaged. Therefore, the colour-changing effect of the thermochromic ink is substantially unaffected and still has a colour-changing function. When the temperature of the finish film rises, the thermochromic ink undergoes electron transfer [22]. When the temperature is lowered, the thermochromic ink changes to the original colour.

## 4. Conclusions

This study shows that the thermochromic ink concentrations of 15.0% and 30.0% are better and that the gloss of the finish film does not have a linear relationship with its concentration. The gloss reaches the maximum when the thermochromic ink concentration is 10.0%, and it is the lowest when the concentration is 30.0%. The thermochromic ink concentration does not affect the adhesion of the finish film. When the concentration exceeds 15.0%, the concentration is positively related to the impact resistance of the finish film. The concentration is not related to the liquid resistance of the finish film. The thermochromic ink concentration has no effect on the liquid resistance level of the finish film. When the concentration is 15.0%, the particles are evenly distributed. The finnish film on Cunninghamia lanceolata has better discoloration effects and good stability when the concentration is 15.0%. This work provides a new prospect for thermochromic waterborne finish films on the surface of Cunninghamia lanceolata.

## Figures and Tables

**Table 1:** The composition of finish coating with thermochromic ink.

Thermochromic Ink Concentration (%)	Thermochromic Ink Weight (g)	Waterborne Finish Weight (g)	Thermochromic Finish Weight (g)
0	0	50.0	50.0
5.0	2.5	47.5	50.0
10.0	5.0	45.0	50.0
15.0	7.5	42.5	50.0
20.0	10.0	40.0	50.0
25.0	12.5	37.5	50.0
30.0	15.0	35.0	50.0

**Table 2:** Effect of thermochromic ink concentration on the gloss of finish film.

Thermochromic Ink Concentration (%)	20° Gloss (%)	60° Gloss (%)	85° Gloss (%)
0	14.1 ± 0.4	43.1 ± 0.5	54.9 ± 1.6
5.0	14.3 ± 0.6	43.8 ± 0.5	54.7 ± 1.3
10.0	19.9 ± 0.7	50.6 ± 0.6	59.7 ± 1.8
15.0	18.6 ± 0.6	43.8 ± 0.5	52.1 ± 1.6
20.0	18.6 ± 0.3	50.1 ± 1.5	59.5 ± 1.5
25.0	13.4 ± 0.1	42.1 ± 0.4	52.6 ± 1.4
30.0	8.4 ± 0	33.5 ± 0.3	44.9 ± 1.2

**Table 3:** Effect of thermochromic ink concentration on the adhesion and impact resistance of finish film.

Sample	0	5.0%	10.0%	15.0%	20.0%	25.0%	30.0%
Damaged area (%)	0 ± 0	0 ± 0	0 ± 0	0 ± 0	0 ± 0	0 ± 0	0 ± 0
Adhesion level (level)	0 ± 0	0 ± 0	0 ± 0	0 ± 0	0 ± 0	0 ± 0	0 ± 0
Impact resistance (N cm^−1^)	40.0 ± 0.5	30.0 ± 0.4	40.0 ± 0.5	40.0 ± 0.5	50.0 ± 0.6	50.0 ± 0.6	70.0 ± 0.7

**Table 4:** Effect of thermochromic ink concentration on colour diference of waterborne finish film for liquid resistance.

Thermochromic Ink Concentration (%)	After Experiment of NaCl	After Experiment of Detergent	After Experiment of Ethanol	After Experiment of Red Ink
0	0.8 ± 0	0.8 ± 0	1.0 ± 0	1.9 ± 0
5.0	1.1 ± 0	1.1 ± 0	1.0 ± 0	1.9 ± 0
10.0	1.8 ± 0	0.5 ± 0	0.7 ± 0	1.3 ± 0
15.0	1.0 ± 0	0.7 ± 0	1.2 ± 0	1.2 ± 0
20.0	0.9 ± 0	1.0 ± 0	1.0 ± 0	1.3 ± 0
25.0	1.2 ± 0	1.3 ± 0	1.0 ± 0	1.2 ± 0
30.0	0.7 ± 0	1.5 ± 0	1.3 ± 0	1.2 ± 0

**Table 5:** Coating liquid resistance level.

Level	Finish Film Change
1	No mark
2	Slightly discolored impression
3	Slight discoloration or noticeable discoloration
4	Obvious changes, bubbling, wrinkles, etc.

**Table 6:** Effect of thermochromic ink concentration on liquid resistance level of finish film.

Thermochromic Ink Concentration (%)	NaCl	Detergent	Ethanol	Red Ink
0	1 ± 0	1 ± 0	1 ± 0	1 ± 0
5.0	1 ± 0	1 ± 0	1 ± 0	1 ± 0
10.0	1 ± 0	1 ± 0	1 ± 0	1 ± 0
15.0	1 ± 0	1 ± 0	1 ± 0	1 ± 0
20.0	1 ± 0	1 ± 0	1 ± 0	1 ± 0
25.0	1 ± 0	1 ± 0	1 ± 0	1 ± 0
30.0	1 ± 0	1 ± 0	1 ± 0	1 ± 0

**Table 7:** Effect of time on colour difference of thermochromic waterborne finish coating.

Thermochromic Ink Concentration (%)	Room Temperature Difference after Three Months	30 °C 24 h Colour Difference after Three Months	30 °C 48 h Colour Difference after Three Months	30 °C 72 h Colour Difference after Three Months
15.0	1.3 ± 0	1.7 ± 0	1.6 ± 0	1.4 ± 0

**Table 8:** Colour difference and gloss change after UV resistance.

Sample	L*	a*	b*	C	H	ΔE	60° Gloss (%)
**Before aging**	67.4 ± 0.4	37.2 ± 0.2	26.9 ± 0.1	45.9 ± 0.3	35.9 ± 0.2	-	43.8 ± 0.3
**After aging**	65.8 ± 0.8	35.2 ± 0.2	29.3 ± 0.1	45.8 ± 0.4	39.8 ± 0.2	3.5 ± 0.1	41.9 ± 0.8

**Table 9:** Assignment of the bands.

Band (cm^−^^1^)	Assignment
3383, 3343	–OH absorption
2931, 2852	–CH_2_– asymmetric and symmetric stretching vibrations
2931, 2852, 1446	–CH_3_ absorption
1600	C=C, N=N absorption
1729	C=O absorption
